# Effects of concurrent cartilage procedures on cartilage regeneration in high tibial osteotomy: a systematic review

**DOI:** 10.1186/s43019-024-00221-w

**Published:** 2024-03-28

**Authors:** Joo Hyung Han, Min Jung, Kwangho Chung, Se-Han Jung, Chong-Hyuk Choi, Sung-Hwan Kim

**Affiliations:** 1 Department of Orthopedic Surgery, Yonsei University College of Medicine, Seoul, Republic of Korea; 2 Department of Orthopedic Surgery, Arthroscopy and Joint Research Institute, Yonsei University College of Medicine, Seoul, Republic of Korea; 3 Department of Orthopedic Surgery, Severance Hospital, Yonsei University College of Medicine, Seoul, Republic of Korea; 4 Department of Orthopedic Surgery, Yongin Severance Hospital, Yonsei University College of Medicine, Yongin, Republic of Korea; 5 Department of Orthopedic Surgery, Gangnam Severance Hospital, Yonsei University College of Medicine, 211 Eonju-Ro, Gangnam-Gu, Seoul, 130-729 Korea

**Keywords:** High tibial osteotomy, Human umbilical cord blood-derived mesenchymal stem cell, Bone marrow aspirate concentrate, Microfracture, Cartilage regeneration, Second-look arthroscopy

## Abstract

**Purpose:**

This systematic review aimed to evaluate the effects of concurrent cartilage procedures on cartilage regeneration when performed alongside high tibial osteotomy (HTO).

**Materials and methods:**

The systematic review followed the guidelines outlined in the Preferred Reporting Items for Systematic Reviews and Meta-Analysis (PRISMA). A comprehensive search was conducted on databases including PubMed, Embase, Cochrane Library, and Google Scholar, covering articles published until August 31, 2023.

**Results:**

Sixteen studies (1277 patients) revealed that HTO, with or without concurrent cartilage procedures, leads to cartilage regeneration based on the International Cartilage Repair Society (ICRS) grade during second-look arthroscopy. No concurrent procedure showed improvement in ICRS grade (mean difference: − 0.80 to − 0.49). Microfracture (mean difference: − 0.75 to − 0.22), bone marrow aspirate concentrate (BMAC) (mean difference: − 1.37 to − 0.67), and human umbilical cord blood-derived mesenchymal stem cell (hUCB-MSC) (mean difference: − 2.46 to − 1.81) procedures also demonstrated positive outcomes. Clinical outcome assessments for each cartilage procedure were also improved during postoperative follow-up, and no specific complications were reported.

**Conclusions:**

HTO with or without concurrent cartilage procedures promotes cartilage regeneration observed during second-look arthroscopy, with improved clinical outcomes. Future randomized controlled trials on the same topic, along with subsequent meta-analyses, are necessary for conclusive findings.

**Supplementary Information:**

The online version contains supplementary material available at 10.1186/s43019-024-00221-w.

## Introduction

High tibial osteotomy (HTO) is a surgical procedure often used to treat unicompartmental knee osteoarthritis, particularly when realigning the knee joint becomes necessary [1–3]. This procedure involves modifying the alignment of the tibial plateau to reduce excessive load on the affected joint compartment, thereby alleviating pain and potentially slowing osteoarthritis progression [4–6]. Recently, the combination of HTO and concurrent cartilage procedures has gained considerable attention in orthopedic surgery [7, 8].

Concurrent cartilage procedures performed alongside HTO primarily aimed to enhance cartilage regeneration and overall joint preservation. These procedures involve several techniques. Subchondral drilling (SD) or microfracture (MFX) involves the creation of small holes or fractures in the subchondral bone beneath the articular cartilage [9, 10]. These processes stimulate the release of bone marrow cells and growth factors, fostering fibrocartilage formation in the damaged areas. These outcomes may be attributed to insufficient stimulation of function and a lower number of recruited mesenchymal stem cells (MSCs) [11, 12].

In bone marrow aspirate concentrate (BMAC) augmentation, bone marrow is extracted, stem cells and growth factors are concentrated, and the resulting mixture is used to facilitate cartilage repair [13]. BMAC augmentation depends on the inclusion of various growth factors and pluripotent stromal cells that induce MSCs differentiation into chondrocytes [14]. This process potentially produces native, hyaline-like cartilage.

Human umbilical cord blood-derived mesenchymal stem cells (hUCB-MSCs) utilize stem cells from human umbilical cord blood and offer potential contributions to cartilage repair and regeneration when introduced into the knee joint [15]. hUCB-MSCs are recognized for their low immunogenicity and the convenience of being a non-invasive collection method. Furthermore, they demonstrate a robust expansion capacity, ensuring a sufficient cell supply for effective treatment [16].

Based on previously published meta-analyses and systematic reviews, Park et al. [17] conducted a meta-analysis comparing cartilage regeneration and clinical scores between BMAC and hUCB-MSC therapies when performed with HTO. Lee et al. [7] and Kehlenberg et al. [18] performed meta-analyses of the clinical effects of concurrent cartilage procedures performed with HTO. To the best of our knowledge, this is the first meta-analysis and systematic review investigating the second-look arthroscopy results for all types of concurrent cartilage procedures performed alongside HTO. This systematic review and meta-analysis aimed to critically evaluate the effect of concurrent cartilage procedures on cartilage regeneration when performed alongside HTO. By synthesizing evidence from relevant studies, we provide a comprehensive perspective on the efficacy of these concurrent procedures in enhancing cartilage regeneration and improving clinical outcomes following HTO.

## Methods

### Search strategy

The review was registered a priori in the PROSPERO prospective register of systematic reviews (ID: CRD42023474067) and conducted according to a predefined protocol and in line with the Preferred Reporting Items for Systematic Reviews and Meta-Analyses (PRISMA) guidelines. A comprehensive search strategy was devised to identify the relevant studies. We systematically searched PubMed, Embase, Cochrane Library, and Google Scholar for articles published until August 31, 2023. The search terms used were [(“HTO” OR “high tibial osteotomy” OR “proximal tibial osteotomy”) AND ((“second look” OR “second-look”) AND “arthroscopy”) AND “knee” AND “osteoarthritis”[mesh]].

### Identification of eligibility

Two independent reviewers screened the search results to determine eligibility. The inclusion criteria were as follows: (1) adult patients diagnosed with OA; (2) studies that included interventions, such as HTO with concurrent cartilage procedures; (3) studies that reported the results of second-look arthroscopy; and (4) studies with a minimum follow-up period of 12 months. The exclusion criteria were as follows: (1) non-English articles; (2) studies with incomplete data; and (3) studies that did not meet the aforementioned criteria.

For conducting a high-quality systematic review, it is desirable to include studies with a high level of evidence. However, pilot search results revealed a limited number of randomized controlled trials addressing the topic, with only one identified. Consequently, to draw appropriate conclusions, the inclusion criteria were set as outlined above. For studies conducted by the same study group where patient groups were expected to overlap, only studies with a higher level of evidence or lower bias risk were included. Two independent reviewers screened the search results to determine eligibility.

### Data extraction

Two reviewers independently extracted data, including the first author, publication year, study design, level of evidence, type of osteotomy, type of concurrent cartilage procedure, sex, age, body mass index, sample size, preoperative International Cartilage Repair Society (ICRS) grade, mean follow-up duration, clinical assessments, postoperative ICRS-Cartilage Repair Assessment grade, postoperative Koshino stage, postoperative histological assessments, postoperative magnetic resonance observation of cartilage repair tissue (MOCART) score, and reported complications. Data pooling for the cartilage regeneration assessment was conducted with a focus on the outcomes of the medial compartment, which was the target of the cartilage procedure.

To assess the risk of bias, we used the methodological index for non-randomized studies (MINORS), consisting of 12 categories for comparative studies and eight categories for non-comparative studies. Each category received a rating of 0 (if not reported), 1 (if reported but inadequate), or 2 (if reported and deemed adequate). Quality of non-randomized-controlled trials was evaluated by the Risk of Bias in Non-randomized Studies of Interventions (ROBINS-I) tool.

### Statistical analysis

Statistical analyses were conducted using the appropriate meta-analysis techniques. Descriptive statistics, including the mean and standard deviation for numerical variables, were recorded. In cases where the studies did not provide a standard deviation in their results, we calculated it based on other provided statistical values, following the method outlined by Furukawa et al. [19]. For the analysis of continuous outcome measures of the assessment of cartilage using the ICRS grade, we utilized the mean differences (MD) with 95% confidence intervals (CIs). We based our analysis on the ICRS grade as the primary outcome for cartilage regeneration. A subgroup meta-analysis was attempted for treatment approaches reported in two or more studies. Heterogeneity was assessed using the I^2^ statistic. I^2^ was calculated after the inclusion of subsequently poorer quality studies in a cumulative meta-analysis for a sensitivity analysis. The subgroups used in the analysis included no concurrent procedure, MFX, BMAC, and hUCB-MSC. Qualitative comparisons were made between the data, and pooling was avoided due to heterogeneity between included studies. Only the results of studies included in this review were presented using forest plots. All statistical analyses and data visualization were performed using R software (version 4.2.1; R Foundation, Vienna, Austria).

## Results

### Characteristics of included studies

**Fig. 1:**
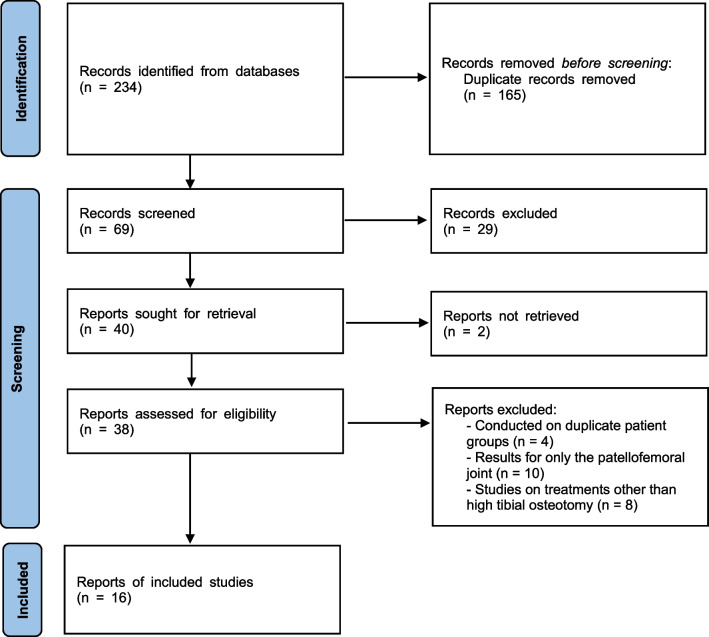
PRISMA flow diagram for the systematic review

In this systematic review and meta-analysis, 234 relevant studies were identified from various databases. After removing duplicates and reviewing the full texts, 38 studies were evaluated for eligibility. Ultimately, we included 16 studies with 1277 patients who met our inclusion criteria (Fig. 1) [20–34]. Mean follow-up period of the included studies ranged from 1.0 year to 3.0 years. The characteristics of the included studies are shown in Tables 1 and 2.

**Table 1:** Overview of included studies

Study	Year	Journal	Country	Study design	Level of evidence	Type of osteotomy	Cartilage procedures	Number of patients
Shon et al. [20]	2023	*Arthroscopy*	South Korea	Retrospective comparative study	3	MOW	SD versus SD + PCHCA	102
Kim et al. [21]	2023	*Arthrosc Sports Med Rehabil*	South Korea	Retrospective comparative study	3	MOW	SVF versus hUCB-MSC	50
Wu et al. [22]	2023	*J Orthop Surg (Hong Kong)*	China	Retrospective case series	4	Not mentioned	No procedure	50
Park et al. [23]	2023	*Medicina (Kaunas)*	South Korea	Retrospective case series	4	MOW	hUCB-MSC	12
Lee et al. [24]	2023	*Biomedicines*	South Korea	Retrospective case series	4	MOW	No procedure	65
Yang et al. [25]	2022	*Knee Surg Sports Traumatol Arthrosc*	South Korea	Retrospective comparative study	3	MOW	BMAC versus hUCB-MSC	110
Kim et al. [34]	2022	*Orthop J Sports Med*	South Korea	Cohort study	3	MOW	No procedure	122
Otsuki et al. [26]	2022	*Cartilage*	Japan	Therapeutic case series	4	MOW	No procedure	142
Jin et al. [32]	2021	*Knee Surg Sports Traumatol Arthrosc*	South Korea	Retrospective comparative study	3	MOW	MFX versus BMAC	91
Chung et al. [27]	2021	*Int Orthop*	South Korea	Retrospective case series	4	MOW	hUCB-MSC	93
Iida et al. [28]	2021	*J Exp Orthop*	Japan	Therapeutic case series	4	MOW	MFX	8
Song et al. [29]	2020	*World J Stem Cells*	South Korea	Retrospective case series	4	MOW	hUCB-MSC	125
Lee et al. [30]	2019	*BMC Musculoskelet Disord*	South Korea	Retrospective case-control study	3	MOW	MFX versus no procedure	87
Kim et al. [35]	2017	*Am J Sports Med*	South Korea	Randomized controlled trial	2	MOW	MFX versus MFX + collagen	28
Kumagai et al. [31]	2017	*Knee Surg Sports Traumatol Arthrosc*	Japan	Retrospective case series	4	MOW	No procedure	131
Jung et al. [33]	2015	*Arthroscopy*	South Korea	Retrospective comparative study	3	MOW	SD versus no procedure	61

*MOW* medial open wedge, *SD* subchondral drilling, *PCHCA* particulated costal hyaline cartilage allograft, *SVF* stromal vascular fraction, *hUCB-MSC* human umbilical cord blood-derived mesenchymal stem cell, *BMAC* bone marrow aspirate concentrate, *MFX* microfracture

**Table 2:** Patient demographics of included studies

Study	Follow-up duration	Sex (n, male/female)	Age (years)	BMI (kg/m^2^)
Shon et al. 2023 [20]	26.2 ± 8.1 months (SD), 27.9 ± 8.7 months (SD + PCHCA)	7/44 (SD), 9/42 (SD + PCHCA)	55.8 ± 5.3 (SD), 55.3 ± 5.5 (SD + PCHCA)	26.9 ± 3.5 (SD), 26.3 ± 3.5 (SD + PCHCA)
Kim et al. 2023 [21]	27.8 ± 3.6 months (SVF), 28.2 ± 4.1 months (hUCB-MSC)	8/17 (SVF), 9/16 (hUCB-MSC)	56.0 ± 4.8 (SVF), 56.4 ± 6.0 (hUCB-MSC)	26.1 ± 2.9 (SVF), 26.5 ± 2.7 (hUCB-MSC)
Wu et al. 2023 [22]	14.6 ± 5.8 months	11/39	56 ± 3.4	23.4 ± 3.8
Park et al. 2023 [23]	2.9 years (range 1–6 years)	3/9	54.3 ± 7.8	25.9 ± 2.8
Lee et al. 2023 [24]	26.5 ± 9.1 months	21/44	58 ± 9	27.5 ± 3.1
Yang et al. 2022 [25]	34.2 ± 8.4 months (BMAC), 31.0 ± 6.0 months (hUCB-MSC)	17/38 (BMAC), 13/42 (hUCB-MSC)	55.0 ± 7.3 (BMAC), 56.4 ± 5.3 (hUCB-MSC)	27.2 ± 3.9 (BMAC), 26.8 ± 3.2 (hUCB-MSC)
Kim et al. 2022 [34]	26.0 ± 8.7 months (RKL), 26.1 ± 8.5 months (no RKL)	3/14 (RKL), 11/94 (no RKL)	55.1 ± 4.3 (RKL), 56.1 ± 5.1 (no RKL)	25.8 ± 1.9 (RKL), 25.9 ± 2.7 (no RKL)
Otsuki et al. 2022 [26]	31.0 ± 9.1 months	59/83	63.2 ± 9.6	25.3 ± 4.3
Jin et al. 2021 [32]	36.5 ± 8.2 months (MFX), 33.6 ± 6.6 months (BMAC)	13/30 (MFX), 11/37 (BMAC)	55.8 ± 4.4 (MFX), 56.9 ± 6.1 (BMAC)	25.8 ± 2.9 (MFX), 25.8 ± 3.1 (BMAC)
Chung et al. 2021 [27]	1.7 years (range 1.0–3.5 years)	not mentioned	56.6 (range 43–65)	25.8 (range 20.9–33.2)
Iida et al. 2021 [28]	14.1 ± 4.5 months	0/8	57.6 ± 5.2	26.8 ± 1.8
Song et al. 2020 [29]	3.0 years	95/30	58.3 ± 6.8	25.6 ± 2.7
Lee et al. 2019 [30]	2.0 ± 0.2 years (MFX), 1.9 ± 0.1 years (no procedure)	37/20 (MFX), 7/23 (no procedure)	57.0 ± 5.4 (MFX), 57.0 ± 6.5 (no procedure)	26.5 ± 3.6 (MFX), 26.4 ± 3.3 (no procedure)
Kim et al. 2017 [35]	1.0 year	0/14 (MFX), 1/13 (MFX + collagen)	55.7 ± 5.9 (MFX), 55.4 ± 4.8 (MFX + collagen)	24.1 ± 2.8 (MFX), 24.4 ± 2.7 (MFX + collagen)
Kumagai et al. 2017 [31]	20.8 ± 6.5 months	30/70	66.1 ± 7.7	24.9 ± 3.3
Jung et al. 2015 [33]	25.7 ± 8.3 months (SD), 24.1 ± 5.7 months (no procedure)	3/27 (SD), 3/28 (no procedure)	61.5 ± 7.5 (SD), 58.6 ± 6.9 (no procedure)	25.8 ± 2 (SD), 25.6 ± 2.3 (no procedure)

*MOW* medial open wedge, *SD* subchondral drilling, *PCHCA* particulated costal hyaline cartilage allograft, *SVF* stromal vascular fraction, *hUCB-MSC* human umbilical cord blood-derived mesenchymal stem cell, *BMAC* bone marrow aspirate concentrate, *RKL* radiological kissing lesion, *MFX* microfracture

### Methodological quality assessment of included studies

We assessed the methodological quality of the selected studies, identifying different levels. One study was at level 2 [35], eight at level 3 [20, 21, 25, 30, 32–34], and nine at level 4 [22–24, 26–29, 31]. For comparative studies, the average MINORS score was 19.4 ± 1.4, based on the data from eight studies. Non-comparative studies had an average MINORS score of 10.5 ± 1.6 across eight studies. Additional file 1: Table S1 provides further details on the MINORS scores. The quality of non-randomized controlled trials was assessed using the ROBINS-I tool, and only one study was rated as having an overall low risk of bias. The assessment results using the ROBINS-I tool are presented in Additional file 1: Table S2.

### ICRS grade

**Fig. 2:**
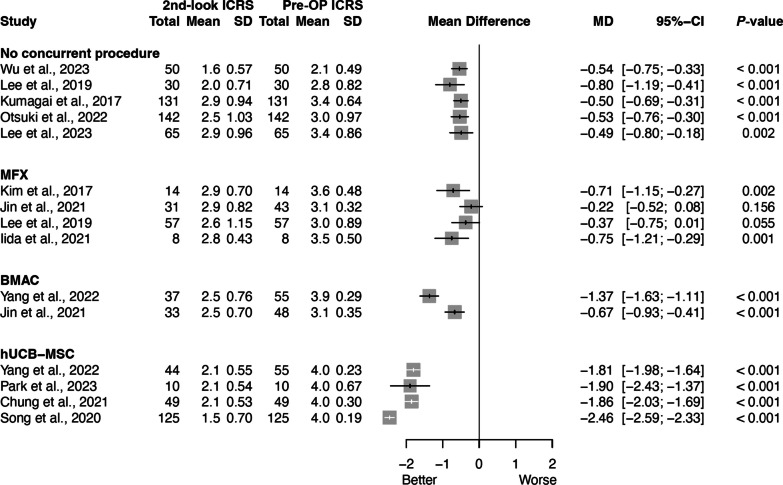
Forest plot of effects of concurrent cartilage procedures based on the International Cartilage Repair Society (ICRS) grade of second-look arthroscopy. *Pre-OP* preoperative, *SD* standard deviation, *MD* mean difference, *CI* confidence interval, *MFX* microfracture, *BMAC* bone marrow aspirate concentrate, *hUCB-MSC* human umbilical cord blood-derived mesenchymal stem cell

The results of preoperative and second-look arthroscopy ICRS grade were reported in 13 studies (Table 3) [20, 22–32, 35]. We attempted a meta-analysis on the ICRS grade results from preoperative and second-look arthroscopy assessments. Substantial heterogeneity was observed (I^2^ = 94%, τ^2^ = 1.4972, *P* < 0.001). A sensitivity analysis was conducted to evaluate whether the inclusion of lower quality studies significantly impacted the heterogeneity of the meta-analyses (Additional file 1: Table S3). However, this analysis resulted in only minimal changes in the I^2^ statistic. Therefore, data pooling was not performed, and the degree of cartilage regeneration in second-look arthroscopy for each cartilage procedure was presented in a forest plot (Fig. 2). Studies were grouped into subcategories based on the treatment methods: no concurrent procedure, MFX, BMAC, and hUCB-MSC.

**Table 3:** Assessment of cartilage regeneration in included studies

Study	ICRS grade		Post-OP Koshino Staging	Post-OP MOCART score	Post-OP histology
	Pre-OP	Post-OP			
Shon et al. 2023 [20]	SD + PCHCA, III (4), IV (47); SD, III (6), IV (45)	SD + PCHCA, I (7), II (36), III (8), IV (7); SD, I (1), II (15), III (21), IV (14)	SD + PCHCA, A (0), B (12), C (39); SD, A (4), B (41), C (6)	N/A	N/A
Kim et al. 2023 [21]	N/A	N/A	N/A	N/A	N/A
Wu et al. 2023 [22]	I (3), II (37), III (10)	I (22), II (26), III (2)	N/A	N/A	increased expression of p-ERK 1/2
Park et al. 2023 [23]	IV (10)	I (1), II (7), III (2)	N/A	N/A	N/A
Lee et al. 2023 [24]	I (3), II (7), III (16), IV (39)	I (7), II (12), III (26), IV (20)	N/A	N/A	N/A
Yang et al. 2022 [25]	BMAC, III (5), IV (50); hUCB-MSC, III (3), IV (52)	BMAC, I (1), II (20), III (11), IV (5); hUCB-MSC, I (4), II (30), III (10)	BMAC, A (4), B (12), C (21); hUCB-MSC, B (12), C (32)	N/A	N/A
Kim et al. 2022 [34]	N/A	N/A	A (25), B (63), C (34)	N/A	N/A
Otsuki et al. 2022 [26]	0-I (10), II (34), III (38), IV (60)	0-I (30), II (37), III (47), IV (28)	N/A	N/A	N/A
Jin et al. 2021 [32]	MFX, III (38), IV (5); BMAC, III (41), IV (7)	MFX, II (12), III (10), IV (9); BMAC, I (1), II (18), III (11), IV (3)	MFX, A (5), B (16), C-1 (9), C-2 (1); BMAC, A (2), B (15), C-1 (14), C-2 (2)	N/A	N/A
Chung et al. 2021 [27]	IV (49)	I (4), II (34), III (11)	B (12), C-1 (27), C-2 (10)	N/A	N/A
Iida et al. 2021 [28]	III (4), IV (4)	II (2), III (6)	N/A	69.2 ± 10.1	N/A
Song et al. 2020 [29]	IV (125)	I (73), II (37), III (15)	N/A	N/A	N/A
Lee et al. 2019 [30]	MFX, I (3), II (13), III (21), IV (20); No procedure, I (2), II (7), III (15), IV (6)	MFX, 0 (3), I (8), II (9), III (23), IV (14); No procedure, I (7), II (15), III (8)	N/A	41.8 ± 18.6 (MFX), 31.8 ± 19.8 (No procedure)	N/A
Kim et al. 2017 [35]	MFX, III (5), IV (9); MFX + Collagen, III (6), IV (8)	MFX, II (4), III (7), IV (3); MFX + Collagen, I (3), II (9), III (2)	N/A	45.4 ± 11.5 (MFX), 64.6 ± 14.1 (MFX + Collagen)	ICRS-II score 885.4 (MFX), 1053.2 (MFX + Collagen)
Kumagai et al. 2017 [31]	II (11), III (53), IV (67)	I (14), II (21), III (56), IV (40)	N/A	N/A	N/A
Jung et al. 2015 [33]	N/A	N/A	N/A	N/A	N/A

*ICRS* International Cartilage Repair Society, *Pre-OP* preoperative, *Post-OP* postoperative, *MOCART* magnetic resonance observation of cartilage repair tissue, *SD* subchondral drilling, *PCHCA* particulated costal hyaline cartilage allograft, *N/A* not applicable, *hUCB-MSC* human umbilical cord blood-derived mesenchymal stem cell, *BMAC* bone marrow aspirate concentrate, *MFX* microfracture

The results for the no concurrent procedure were reported in five studies [22, 24, 26, 30, 31], showing an improvement in ICRS grade with a mean difference ranging from − 0.80 to − 0.49 between the preoperative and second-look time points. For MFX, the results from four studies indicated a mean difference ranging from − 0.75 to − 0.22 [28, 30, 32, 35]. BMAC results were reported in two studies, with a mean difference range of − 1.37 to − 0.67 [25, 32]. The hUCB-MSC results, reported in four studies, showed a mean difference range of − 2.46 to − 1.81 [23, 25, 27, 29].

Additionally, Shon et al. [20] reported that SD with particulated costal hyaline cartilage allograft (PCHCA) showed a significantly better cartilage status compared to subchondral drilling alone based on the ICRS-CRA grading system (*P* < 0.001). Kim et al. [35] compared the results of MFX alone with MFX with collagen augmentation. In the MFX with collagen augmentation group, three patients (21.4%) were classified as normal (grade 1), nine (64.3%) as nearly normal (grade 2), and two (14.3%) as abnormal (grade 3).

### Koshino staging

The results of postoperative Koshino staging were reported in five studies (Table 3) [20, 25, 27, 32, 34]. Shon et al. [20] found that SD with PCHCA resulted in the following stage distribution: stage A (0 cases), stage B (12 cases), and stage C (39 cases). In the SD alone, the distribution was stage A (4 cases), stage B (41 cases), and stage C (6 cases). The findings demonstrated a significant improvement in the Koshino stage with SD and PCHCA (*P* < 0.001).

Yang et al. [25] reported stages A (4 cases), B (12 cases), and C (21 cases) in the BMAC treatment group, and stages A (0 cases), B (12 cases), and C (32 cases) in the hUCB-MSC treatment group. No significant differences were found between the treatments (*P* = 0.057). Jin et al. [32] observed the following stage distribution in the MFX treatment group: stage A (5 cases), stage B (16 cases), stage C-1 (9 cases), and stage C-2 (1 case). In the BMAC treatment group, the distribution was stage A (2 cases), stage B (15 cases), stage C-1 (14 cases), and stage C-2 (2 cases). However, no significant differences were noted between the treatments (*P* = 0.187).

Kim et al. [34] reported 25 cases of stage A, 63 cases of stage B, and 34 cases of stage C in the group without concurrent cartilage procedures performed. Chung et al. [27] found 0 case of stage A, 12 cases of stage B, 27 cases of stage C-1, and 10 cases of stage C-2 in the hUCB-MSC treatment group.

### MOCART score

Postoperative MOCART scores were reported in three studies (Table 3) [28, 30, 35]. Lee et al. [30] reported scores of 41.8 ± 18.6 for the MFX group and 31.8 ± 19.8 for the group without procedure. Significantly higher scores were found in the MFX group than in the no-procedure group (*P* = 0.023). Kim et al. [35] reported scores of 45.4 ± 11.5 for the MFX group and 64.6 ± 14.1 for the MFX with collagen augmentation group. They observed a significantly higher score in the MFX with collagen augmentation group than in the MFX group (*P* = 0.001). Iida et al. [28] reported a score of 69.2 ± 10.1 for the MFX group.

### Histological assessment

Histological assessments were reported in two studies [22, 35]. Wu et al. [22] found that HTO without concurrent cartilage procedures significantly upregulated the expression of p-ERK1/2 at the protein level in patients with knee osteoarthritis compared with that in the control group. Kim et al. [35] reported ICRS II scores of 885.4 for the MFX group and 1053.2 for the MFX with collagen augmentation group. They observed a significantly higher score in the MFX with the collagen augmentation group (*P* = 0.002).

### Clinical outcome assessments

The clinical outcome assessments reported in the included studies, including IKDC score, WOMAC score, and KSS pain and function score, are presented in Table 4. IKDC scores were reported in eight studies [24, 25, 27–29, 32, 34, 35]. The WOMAC scores were reported in five studies [24, 27, 29, 30, 32]. KSS pain and function scores were reported in six studies [27, 30–34]. In all studies, an improvement in clinical outcomes compared to the preoperative period was reported at the follow-up time points.

**Table 4:** Clinical scores in included studies

Study	IKDC score			WOMAC score			KSS-Pain			KSS-Function		
	Pre-OP	Post-OP	*P*-value	Pre-OP	Post-OP	*P*-value	Pre-OP	Post-OP	*P*-value	Pre-OP	Post-OP	*P*-value
Shon et al. 2023 [20]	N/A	N/A	N/A	48.3 ± 12.1 (SD + PCHCA), 48.8 ± 16.1 (SD)	11.2 ± 14.9 (SD + PCHCA), 14.7 ± 19.3 (SD)	< 0.001 (SD + PCHCA), < 0.001 (SD)	N/A	N/A	N/A	N/A	N/A	N/A
Kim et al. 2023 [21]	38.5 ± 4.1 (SVF), 37.9 ± 4.3 (hUCB-MSC)	72.4 ± 6.1 (SVF), 71.8 ± 6.1 (hUCB-MSC)	< 0.001 (SVF), < 0.001 (hUCB-MSC)	N/A	N/A	N/A	N/A	N/A	N/A	N/A	N/A	N/A
Wu et al. 2023 [22]	25.3 ± 10.3	58.2 ± 17.3	< 0.001	61.1 ± 11.8	22.2 ± 8.4	< 0.001	N/A	N/A	N/A	N/A	N/A	N/A
Park et al. 2023 [23]	36.2 ± 3 (BMAC), 35.4 ± 5.5 (hUCB-MSC)	72.8 ± 5 (BMAC), 3.3 ± 9.8 (hUCB-MSC)	N/A	N/A	N/A	N/A	N/A	N/A	N/A	N/A	N/A	N/A
Lee et al. 2023 [24]	N/A	N/A	N/A	N/A	N/A	N/A	83.2 ± 4.8	94 ± 2.4	< 0.001	75 ± 6.3	96.5 ± 6.2	< 0.001
Yang et al. 2022 [25]	N/A	N/A	N/A	43.9 ± 12.7 (BMAC), 45.2 ± 8.8 (hUCB-MSC)	23.4 ± 11.6 (BMAC), 19.5 ± 15.8 (hUCB-MSC)	N/A	30.8 ± 11 (BMAC), 31.6 ± 10.4 (hUCB-MSC)	40.6 ± 9.1 (BMAC), 42.8 ± 7.9 (hUCB-MSC)	N/A	62.3 ± 11.9 (BMAC), 63.1 ± 11.2 (hUCB-MSC)	80.1 ± 15 (BMAC), 82.4 ± 15.5 (hUCB-MSC)	N/A
Kim et al. 2022 [34]	33.7 ± 9.4 (MFX), 35.3 ± 12.6 (BMAC)	67 ± 10.6 (MFX), 71.3 ± 11.2 (BMAC)	< 0.001 (MFX), < 0.001 (BMAC)	47.5 ± 10.4 (MFX), 46.9 ± 13.9 (BMAC)	20.4 ± 9.7 (MFX), 16.3 ± 9.8 (BMAC)	< 0.001 (MFX), < 0.001 (BMAC)	27 ± 8.5 (MFX), 27.2 ± 7.6 (BMAC)	39.7 ± 6.5 (MFX), 42.6 ± 7.2 (BMAC)	< 0.001 (MFX), < 0.001 (BMAC)	60.6 ± 11 (MFX), 58.9 ± 13.3 (BMAC)	88.8 ± 8.2 (MFX), 91 ± 10.2 (BMAC)	< 0.001 (MFX), < 0.001 (BMAC)
Otsuki et al. 2022 [26]	39 ± 10.4	71.3 ± 5.9	< 0.001	44.5 ± 15.1	11 ± 3.7	< 0.001	29.8 ± 11.8	43.2 ± 5	< 0.001	61 ± 16.3	81.2 ± 13.7	< 0.001
Jin et al. 2021 [32]	29 ± 7.4	64.9 ± 11.1	N/A	44.1 ± 10.6	8.4 ± 6.5	N/A	N/A	N/A	N/A	N/A	N/A	N/A
Chung et al. 2021 [27]	N/A	N/A	N/A	39.8 ± 13.2 (MFX), 39.5 ± 7.8 (No procedure)	9.2 ± 6.1 (MFX), 10.2 ± 7.8 (No procedure)	N/A	53.7 ± 17 (MFX), 52.7 ± 14.3 (No procedure)	89.1 ± 10.7 (MFX), 88.3 ± 10.8 (No procedure)	N/A	59.5 ± 15.5 (MFX), 59.8 ± 9 (No procedure)	88.3 ± 10.8 (MFX), 86.1 ± 12.3 (No procedure)	N/A
Iida et al. 2021 [28]	38.7 ± 14.3 (MFX), 33.4 ± 14.6 (MFX + Collagen)	60.3 ± 10.1 (MFX), 57.2 ± 13.6 (MFX + Collagen)	N/A	N/A	N/A	N/A	N/A	N/A	N/A	N/A	N/A	N/A
Song et al. 2020 [29]	N/A	N/A	N/A	N/A	N/A	N/A	49.7 ± 12.2	87.6 ± 9.2	N/A	65.1 ± 14.7	92.3 ± 11	N/A
Lee et al. 2019 [30]	N/A	N/A	N/A	N/A	N/A	N/A	67.3 ± 8.2 (SD), 63.7 ± 13.9 (No procedure)	91.2 ± 6.4 (SD), 92.5 ± 5.3 (No procedure)	N/A	66.5 ± 14.3 (SD), 66.8 ± 9.1 (No procedure)	92.8 ± 10 (SD), 92.2 ± 8 (No procedure)	N/A
Kim et al. 2017 [35]	N/A	N/A	N/A	N/A	N/A	N/A	68.5 ± 11.9	92.5 ± 7.1	0.001	62.3 ± 11.4	90.4 ± 9	0.001

*IKDC* International Knee Documentation Committee, *WOMAC* Western Ontario and McMaster Universities Arthritis Index, *KSS* Knee Society score, *Pre-OP* preoperative, *Post-OP* postoperative, *MOCART* magnetic resonance observation of cartilage repair tissue, *SD* subchondral drilling, *PCHCA* particulated costal hyaline cartilage allograft, *N/A* not applicable, *SVF* stromal vascular fraction, *hUCB-MSC* human umbilical cord blood-derived mesenchymal stem cell, *BMAC* bone marrow aspirate concentrate, *MFX* microfracture

### Complications

Postoperative complications were reported in four studies [22, 25, 27, 34]. Yang et al. [25] reported that among 55 patients who underwent the BMAC procedure, one patient experienced postoperative stiffness. The patient underwent manipulation under anesthesia 2 months postoperatively and recovered without undergoing any further procedures. Kim et al. [34] reported that among 122 patients who did not undergo concurrent cartilage procedures, two patients required revisional HTO due to the early collapse of the opening gap. Additionally, one patient developed a late hematogenous infection 15 months postoperatively after dental treatment. The patient underwent plate removal and debridement. Wu et al. [22] reported no complications in 50 patients who did not undergo concurrent cartilage procedures. Chung et al. [27] reported no complications in 93 patients who underwent the hUCB-MSC procedure.

## Discussion

This systematic review aimed to critically assess the effect of concurrent cartilage procedures when performed alongside HTO. It included 16 studies involving 1277 patients. The key finding of this study lies in comprehensively examining the effectiveness of each cartilage procedure by investigating all studies that reported second-look arthroscopy results. The results reported in terms of ICRS grade and Koshino grade were compiled; and although there were variations in the degree of improvement, it was confirmed that all concurrent procedures performed with HTO had an effect on cartilage regeneration. Considering the level of evidence and heterogeneity in the studies included in this review, it was deemed inappropriate to compare the overall treatment effect of each procedure through data pooling, and thus, this was not conducted.

In previous meta-analyses and systematic reviews focusing on concurrent cartilage procedures performed alongside HTO, Park et al. [23] analyzed the ICRS grade for BMAC and hUCB-MSC therapies and reported significantly superior cartilage regeneration in the hUCB-MSC group. Lee et al. [7] noted that one study found no significant differences in fibrocartilage formation between the HTO-only and HTO-plus-arthroscopic drilling groups [33]. In studies involving concurrent abrasion arthroplasty and human autologous culture-expanded bone marrow mesenchymal cell transplantation, the control groups exhibited more favorable healing outcomes compared to the case groups [36].

The MFX technique recruits bone marrow elements to repair cartilage defects. However, it lacks a stable long-term efficacy and is ineffective in the treatment of large cartilage defects [37]. Mithoefer et al. [38] reported that a review of 28 trials confirmed deterioration within 2 years, highlighting limitations such as the absence of hyaline tissue repair, variable cartilage volume restoration, and potential functional decline.

Concentrated MSCs from autologous bone marrow offer an emerging approach for treating cartilage disease [32]. This method simplifies MSC acquisition, enabling the entire process from harvesting to transplantation in a single operation [39]. BMACs contain growth factors that promote cartilage regeneration and MSC adhesion. BMACs also possess immunomodulatory and anti-inflammatory properties that promote cartilage restoration. However, achieving consistent cell numbers and concentrations can be challenging as centrifugation procedures are performed in the operating field.

hUCB-MSCs are derived from the umbilical cord blood and have emerged as a promising treatment for cartilage regeneration. Studies have reported improved outcomes in knee osteoarthritis after hUCB-MSC application [40]. Cells obtained from donors are typically expanded in culture before being injected into the affected joint or used in combination with other procedures. Notably, hUCB-MSCs have higher proliferation rates and more than 1000-fold greater expansion capacity compared to BMACs. This may affect the effectiveness of cartilage regeneration observed during second-look arthroscopy, depending on the chosen treatment approach.

In addition to the ICRS evaluation of cartilage regeneration, when examining other assessments related to cartilage regeneration, radiographical evaluation of cartilage regeneration was reported using MOCART scores. Lee et al. [30] reported a significantly higher MOCART score in the MFX group than in the no-procedure group. Kim et al. [35] also reported a significantly higher score in the MFX with collagen augmentation group than in the MFX-only group. With regard to the histological evaluation, Kim et al. [35] reported a significantly higher ICRS II score in the MFX with collagen augmentation group than in the MFX-only group.

In all the included studies, improvements in postoperative clinical scores were observed compared to preoperative clinical scores. The primary outcome of this study was the degree of cartilage regeneration confirmed during second-look arthroscopy. Additionally, considering the substantial heterogeneity and level of evidence in the clinical scores reported in the studies included in this research, a meta-analysis for these parameters was not conducted. Apart from the improvement noted in postoperative clinical scores compared to preoperative scores, the overall effects of each cartilage process generally yielded mixed results. These findings were in line with previous meta-analyses.

In accordance with the research conducted by Lee et al. [7] which investigated the clinical effects of concurrent cartilage procedures conducted alongside HTO, studies included in their analysis showed no statistically significant differences in clinical results between groups. One study that performed MSC injection as a concurrent procedure reported additional treatment effects on the IKDC, Lysholm, and Tegner scores. HTO patients with MFXs showed worse HSS scores compared to the control group. Similarly, a meta-analysis conducted by Park et al*.* [17] examining the clinical effects of hUCB-MSCs and BMACs reported no significant differences in IKDC, WOMAC, KSS pain, or KSS function between the two groups. Due to the fact that HTO was performed for all patients included in this study, the proportion of the contribution of cartilage procedures to the improvement in clinical outcomes was not clearly defined. This could be clarified through the analysis of results from randomized controlled trials targeting these interventions in the future.

The role of HTO is not only to reduce symptoms in patients with OA but also to slow down the progression to a state requiring total knee arthroplasty (TKA) in end-stage OA [1]. The follow-up period of the studies included in this research ranged from about 1 to 3 years, which generally corresponds to a short term. The review was conducted focusing on the degree of improvement in cartilage regeneration and clinical outcomes during this period. Consequently, there were inherent limitations in verifying the effectiveness of HTO in further delaying the advancement of OA. Thus, in order to assess how well HTO achieves its other primary objective of postponing the need for TKA, it seems essential to review long-term follow-up studies that incorporate survival analysis. Such research, if undertaken, should encompass radiographic evaluations that go beyond mere confirmation of cartilage regeneration, including serial follow-up data such as the KL grade, to monitor OA progression comprehensively.

Cost-effectiveness is also a crucial issue in the selection of treatment methods. Especially, since biologics like stem cells are used in cartilage procedures, the cost can vary significantly depending on the treatment method. While there may be differences between medical institutions, in South Korea, undergoing HTO alone can start at a cost of around 1000 dollars with health insurance applied. In contrast, adding hUCB-MSC therapy can incur additional costs ranging from 5000 to 7000 dollars or more, and national health insurance typically does not apply in such cases. Due to the heterogeneity identified in this study, a clear superiority of concurrent cartilage procedures has not been established. Moreover, considering that significant improvements in cartilage regeneration and clinical outcomes were observed even in cases of HTO without concurrent cartilage procedure included in this study, it can be considered that there are benefits of HTO alone from a cost-effectiveness standpoint.

The limitations of this study should be acknowledged. First, the majority of the included studies were classified as level 3 or 4 evidence. This categorization was unavoidable due to the limited availability of randomized controlled trials focusing on this specific topic, thereby posing a challenge in obtaining more robust data. Second, the presence of high heterogeneity among studies was observed, leading to the decision not to conduct a pooled analysis. If the issues of a lack of high-level studies and significant heterogeneity are resolved, methods such as network meta-analysis could be utilized for comparing and ranking treatment methods. This would serve as a powerful tool for drawing clear conclusions on this topic. Third, our analysis was exclusively centered on concurrent procedures for medial compartment osteoarthritis during HTO. This raises the possibility that changes in other compartments, such as the patellofemoral joint, may influence clinical outcomes. To address this concern, we intend to conduct future studies that explore other joint compartments. Fourth, some studies lacked a control group consisting of patients undergoing HTO alone. This omission may introduce bias when interpreting the results. Finally, as mentioned in the previous discussion, the follow-up periods in the reviewed studies were not sufficiently extended to comprehensively assess long-term clinical outcomes and survival rates.

In conclusion, HTO performed with or without a concurrent cartilage procedure appears to result in cartilage regeneration observed during second-look arthroscopy compared to the initial state. Clinical outcome assessments also showed improvement, and specific complications associated with concurrent cartilage procedures were not reported. The extent of cartilage regeneration confirmed during second-look arthroscopy varied to some degree for each concurrent cartilage procedure. However, considering the heterogeneity and level of evidence in the studies included in this research, a pooled analysis was not conducted to draw definitive conclusions. Future randomized controlled trials on the same topic, along with subsequent meta-analyses, will be necessary to derive conclusive findings.

### Supplementary Information

**Additional file 1. Table S1.** Quality of the studies was assessed using the MINORS score. **Table S2.** Quality of the non-randomized studies according to the Risk of Bias in Non-randomized Studies of Interventions (ROBINS-I) scale. **Table S3.** I^2^ calculated after the inclusion of subsequently poorer quality studies in a cumulative meta-analysis for a sensitivity analysis.

## Data Availability

The datasets during and/or analysed during the current study available from the corresponding author on reasonable request.
