# Supplementary material for: Effects of concurrent cartilage procedures on cartilage regeneration in high tibial osteotomy: a systematic review
Source: Knee Surg Relat Res. 2024 Mar 28;36:13. doi: 10.1186/s43019-024-00221-w (PMC10979569; doi:10.1186/s43019-024-00221-w)
Supplement: Supplementary file 1 — Additional file 1. Table S1. Quality of the studies was assessed using the MINORS score. Table S2. Quality of the non-randomized studies according to the Risk of Bias in Non-randomized Studies of Interventions (ROBINS-I) scale. Table S3. I2 calculated after the inclusion of subsequently poorer quality studies in a cumulative meta-analysis for a sensitivity analysis. [file 43019_2024_221_MOESM1_ESM.docx]

**Supplementary Table 1.** Quality of the studies was assessed using the MINORS score.

MINORS, methodological index for non-randomized studies

| Study | Kim et al.^35)^ | Yang et al.^25)^ | Jin et al.^32)^ | Lee et al.^30)^ | Shon et al.^20)^ | Jung et al.^33)^ | Kim et al.^21)^ | Kim et al.^34)^ | Wu et al.^22)^ | Park et al.^23)^ | Chung et al.^27)^ | Song et al.^29)^ | Kumagai et al.^31)^ | Otsuki et al.^26)^ | Iida et al.^28)^ | Lee et al.^24)^ |
| --- | --- | --- | --- | --- | --- | --- | --- | --- | --- | --- | --- | --- | --- | --- | --- | --- |
| Year | 2017 | 2022 | 2021 | 2019 | 2023 | 2015 | 2023 | 2022 | 2023 | 2023 | 2021 | 2020 | 2017 | 2022 | 2021 | 2023 |
| Level of evidence | 2 | 3 | 3 | 3 | 3 | 3 | 3 | 3 | 4 | 4 | 4 | 4 | 4 | 4 | 4 | 4 |
| 1. A stated aim of the study | 2 | 2 | 2 | 2 | 2 | 2 | 2 | 2 | 2 | 2 | 2 | 2 | 2 | 2 | 2 | 2 |
| 2. Inclusion of consecutive patients | 1 | 2 | 1 | 1 | 2 | 1 | 1 | 1 | 1 | 2 | 2 | 2 | 2 | 2 | 1 | 2 |
| 3. Prospective collection of data | 2 | 0 | 0 | 0 | 0 | 0 | 0 | 0 | 0 | 0 | 0 | 0 | 0 | 0 | 0 | 0 |
| 4. Endpoint appropriate to the study aim | 2 | 2 | 2 | 2 | 2 | 2 | 2 | 2 | 2 | 2 | 2 | 2 | 2 | 2 | 2 | 2 |
| 5. Unbiased evaluation of endpoints | 2 | 2 | 1 | 2 | 2 | 2 | 2 | 2 | 0 | 0 | 2 | 0 | 0 | 2 | 2 | 2 |
| 6. Follow-up period appropriate to the major endpoint | 2 | 2 | 2 | 2 | 2 | 2 | 2 | 2 | 2 | 2 | 2 | 2 | 2 | 2 | 2 | 2 |
| 7. Loss to follow up not exceeding 5% | 1 | 0 | 1 | 1 | 2 | 1 | 1 | 1 | 2 | 1 | 2 | 0 | 2 | 1 | 2 | 2 |
| 8. Prospective calculation of the sample size | 2 | 0 | 2 | 2 | 0 | 2 | 0 | 0 | 0 | 0 | 0 | 0 | 2 | 0 | 0 | 0 |
| 9. A control group having the gold standard intervention | 2 | 2 | 2 | 2 | 2 | 2 | 2 | 2 | - | - | - | - | - | - | - | - |
| 10. Contemporary groups | 2 | 2 | 2 | 2 | 2 | 2 | 2 | 2 | - | - | - | - | - | - | - | - |
| 11. Baseline equivalence of groups | 2 | 2 | 2 | 2 | 2 | 2 | 2 | 2 | - | - | - | - | - | - | - | - |
| 12. Statistical analyses adapted to the study design | 2 | 2 | 2 | 2 | 2 | 2 | 2 | 2 | - | - | - | - | - | - | - | - |
| Total scores | 22 | 18 | 19 | 20 | 20 | 20 | 18 | 18 | 9 | 9 | 12 | 8 | 12 | 11 | 11 | 12 |

**Supplementary Table 2.** Quality of the non-randomized studies according to the Risk of Bias in Non-randomized Studies of Interventions (ROBINS-I) scale.

| Study | Yang et al.^25)^ | Jin et al.^32)^ | Lee et al.^30)^ | Shon et al.^20)^ | Jung et al.^33)^ | Kim et al.^21)^ | Kim et al.^34)^ |
| --- | --- | --- | --- | --- | --- | --- | --- |
| Year | 2022 | 2021 | 2019 | 2023 | 2015 | 2023 | 2022 |
| Level of evidence | 3 | 3 | 3 | 3 | 3 | 3 | 3 |
| Bias due to confounding | Low | High | Unclear | Low | Unclear | High | High |
| Bias in selection of participants into the study | Unclear | Unclear | Unclear | High | High | Unclear | High |
| Bias in classification of interventions | Low | Low | Unclear | Unclear | Unclear | Unclear | Unclear |
| Bias due to deviations from intended intervention | Low | Unclear | Unclear | Low | Low | Low | Unclear |
| Bias due to missing data | High | Unclear | Unclear | Low | Low | Unclear | Low |
| Bias in measurement of outcomes | Unclear | Unclear | Unclear | Unclear | Unclear | Unclear | Unclear |
| Bias in selection of the reported result | Low | Low | Low | Low | Low | Low | Unclear |

**Supplementary Table 3.** I^2^ calculated after the inclusion of subsequently poorer quality studies in a cumulative meta-analysis for a sensitivity analysis

MINORS, Methodological index for non-randomized studies; IKDC, International Knee Documentation Committee; IKDC, International Knee Documentation Committee; WOMAC, Western Ontario and McMaster Universities Arthritis Index; KSS, Knee Society Score; N/A, not applicable due to the limited number of studies for network meta-analysis

| Study | Year | MINORS score | ICRS grade | IKDC score | WOMAC score | KSS-Pain | KSS-Function |
| --- | --- | --- | --- | --- | --- | --- | --- |
| Kim et al.^35)^ | 2017 | 22 | N/A | N/A | N/A | N/A | N/A |
| Lee et al.^30)^ | 2019 | 20 | 0.6523 | N/A | 0.5334 | N/A | N/A |
| Shon et al.^20)^ | 2023 | 20 | 0.6523 | N/A | 0.5334 | N/A | N/A |
| Jung et al.^33)^ | 2015 | 20 | 0.6523 | N/A | 0.5334 | N/A | 0.374 |
| Jin et al.^32)^ | 2021 | 19 | 0.9167 | 0.7676 | 0.7921 | 0.8499 | 0.8195 |
| Yang et al.^25)^ | 2022 | 18 | 0.952 | 0.9598 | 0.7921 | 0.8499 | 0.8195 |
| Kim et al.^21)^ | 2023 | 18 | 0.952 | 0.9556 | 0.7921 | 0.8499 | 0.8195 |
| Kim et al.^34)^ | 2022 | 18 | 0.952 | 0.9556 | 0.7921 | 0.8878 | 0.8928 |
| Chung et al.^27)^ | 2021 | 12 | 0.952 | 0.9467 | 0.7975 | 0.8897 | 0.9183 |
| Kumagai et al.^31)^ | 2017 | 12 | 0.9527 | 0.9467 | 0.7975 | 0.9288 | 0.9081 |
| Lee et al.^24)^ | 2023 | 12 | 0.9498 | 0.9516 | 0.8408 | 0.9288 | 0.9081 |
| Otsuki et al.^26)^ | 2022 | 11 | 0.9482 | 0.9516 | 0.8408 | 0.9228 | 0.9093 |
| Iida et al.^28)^ | 2021 | 11 | 0.9438 | 0.9478 | 0.8408 | 0.9228 | 0.9093 |
| Wu et al.^22)^ | 2023 | 9 | 0.9392 | 0.9478 | 0.8408 | 0.9228 | 0.9093 |
| Park et al.^23)^ | 2023 | 9 | 0.9392 | 0.9478 | 0.8408 | 0.9228 | 0.9093 |
| Song et al.^29)^ | 2020 | 8 | 0.9392 | 0.9427 | 0.8915 | 0.9228 | 0.9093 |
